# Prognostic value of Cardiac Biomarkers in COVID-19 Infection: A Meta-analysis

**DOI:** 10.21203/rs.3.rs-34729/v1

**Published:** 2020-06-13

**Authors:** Desiree Dawson, Paari Dominic, Aakash Sheth, Malak Modi

**Affiliations:** The Department of Medicine and Center of Excellence for Cardiovascular Diseases & Sciences, Louisiana State University Health Sciences Center-Shreveport (LSUHSC-S); The Department of Medicine and Center of Excellence for Cardiovascular Diseases & Sciences, Louisiana State University Health Sciences Center-Shreveport (LSUHSC-S); The Department of Medicine and Center of Excellence for Cardiovascular Diseases & Sciences, Louisiana State University Health Sciences Center-Shreveport (LSUHSC-S); The Department of Medicine and Center of Excellence for Cardiovascular Diseases & Sciences, Louisiana State University Health Sciences Center-Shreveport (LSUHSC-S)

**Keywords:** Cardiac biomarkers, Mortality, Prognosis, Severity

## Abstract

**Background:**

Multiple Biomarkers have recently been shown to be elevated in COVID-19, a respiratory infection with multi-organ dysfunction; however, information regarding the prognostic value of cardiac biomarkers as it relates to disease severity and cardiac injury are inconsistent.

**Research Question:**

The goal of this meta-analysis was to summarize the evidence regarding the prognostic relevance of cardiac biomarkers from data available in published reports.

**Study Design and Methods:**

PubMed was searched from inception through April 2020 for studies comparing median values of cardiac biomarkers in critically ill versus non-critically ill COVID-19 patients, or patients who died versus those who survived. The weighted mean differences (WMD) and 95% confidence interval (CI) between the groups were calculated for each study and combined using a random effects meta-analysis model. The odds ratio (OR) for mortality based on cardiac injury was combined from studies reporting it.

**Results:**

Troponin levels were significantly higher in COVID-19 patients who died or were critically ill versus those who were alive or not critically ill (WMD 0.58, 95% CI 0.42–0.71, p<0.001). Cardiac injury was independently associated with significantly increased odds of mortality (OR 6.641, 95% CI 1.26 – 35.1, p=0.03). No difference in BNP was seen between the two groups. A significant difference in levels of D-dimer was seen in those who died or were critically ill. CK levels were only significantly higher in those who died versus those who were alive (WMD 0.47 95% CI 0.09–0.84, p=0.014).

**Interpretation:**

Cardiac biomarkers add prognostic value to the determination of the severity of COVID-19 and can predict mortality.

## Introduction

The Coronavirus disease 2019 (COVID–19), caused by Severe Acute Respiratory Syndrome Coronavirus–2 (SARS-CoV–2), has affected over 2,402,250 people worldwide and caused 163,097 deaths as of April 21, 2020.^1^ Lung injury and acute respiratory distress syndrome are the most common presentation of COVID–19, but cardiac injury is another grim consequence of this multisystem viral disease. Evidence of an association between other systemic viral infections such as influenza ^2^ and SARS^3^ and cardiovascular adverse events dates back to the 1930s.^4^ Early observational studies in China and reports from The Centers for Disease Control and Prevention (CDC) suggest that patients with pre-existing heart conditions are more likely to develop severe illness from COVID–19.^5^ A recent study^6^ suggests that patients in the ICU are three-fold more likely to have pre-existing cardiovascular conditions than non-ICU patients. Conversely, newer evidence implies new onset myocardial injury caused by COVID–19.^7, 8^ Possible mechanisms of myocardial injury caused by COVID–19, termed acute COVID–19 cardiovascular syndrome (ACovCS), include heightened myocardial demand in response to the stress of infection, inflammatory cytokines creating a thrombogenic environment as the result of platelet activation and endothelial dysfunction, and direct myocardial damage.^9, 10^ However, current evidence linking myocardial injury as measured by cardiac biomarkers to the severity of disease is conflicting, with a few studies^11, 12,13^ suggesting an association but several others showing a lack of association. ^14,15^Given the discrepancy among these reports, it is prudent to investigate the association of cardiac biomarkers with the severity of disease experienced by and the survival or death of COVID–19 patients. Here we present a meta-analysis of 12 observational studies from China to shed further light on this topic.

## Methods

Our meta-analysis was carried out in accordance with the recommendations of the Meta-analysis of Observational Studies in the Epidemiology Group (MOOSE).^16^

## Search strategies

**Figure 1:**
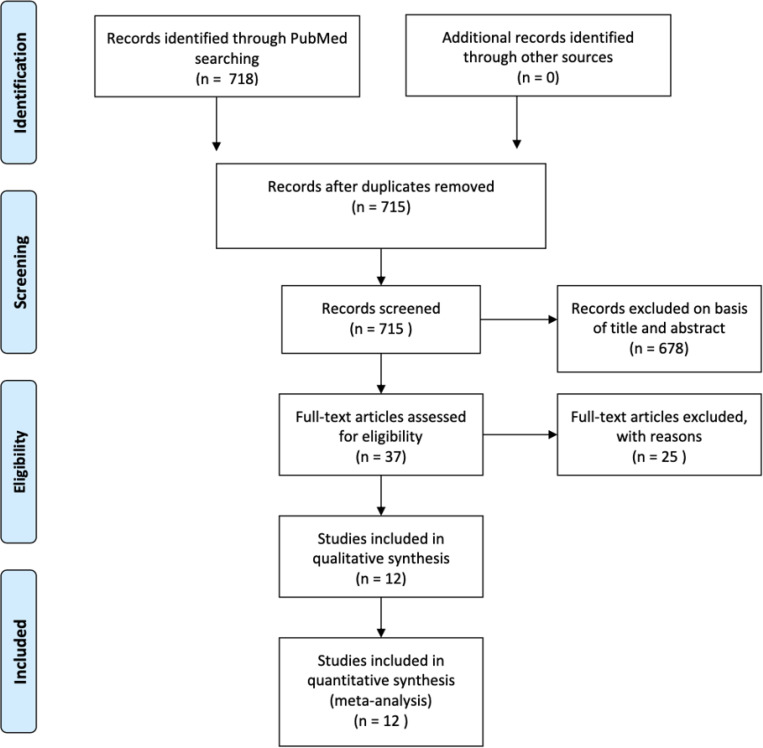
Prisma diagram of literature search and selection.

We searched MEDLINE/PubMed (1966–2020) using the following keywords: (“covid-19” OR “COVID-19” OR “SARS-CoV-2” OR “coronavirus” AND “19”) AND (“Clinical Study” OR “Comparative Study” OR “Evaluation Studies” OR “Meta-Analysis” OR “Multicenter Study” OR “Observational Study” OR “Twin Study” OR “Technical Report” OR “cohort studies” OR “cohort” OR “case-control” OR “cross-sectional” OR “observational” OR “comparative” OR “evaluation”). We also used the ‘Related article’ feature on PubMed, along with a manual search of references, to identify additional studies. We reviewed the full text of all relevant articles. English translations, if necessary, were obtained. Titles and abstracts were independently reviewed by three reviewers (AS, DD, and MM) and cross-verified for inclusion. Details of the search strategy are reported in Figure 1.

## Inclusion criteria

### For the analysis of cardiac biomarker levels in patients with COVID-19

Studies (retrospective and prospective) comparing mean or median troponin and/or BNP in COVID-19 patients who were critically ill versus not critically ill, and those who died versus those who survived, were included. Adult respiratory distress syndrome was considered a surrogate for critical illness and studies that compared findings in COVID-19 patients who developed ARDS to patients without ARDS were included. As these were inhospital outcomes, no specific follow up period was set as an inclusion criteria.

### For the analysis of risk of mortality in COVID-19 patients based on cardiac injury

Studies reporting the risk of mortality with COVID-19 based on the presence of cardiac injury by event rates or univariate or multivariate logistic regression analysis were included. Cardiac injury, for the purpose of this meta-analysis, was defined as an increase in Troponin I, Troponin T, or CK MB. For both groups of studies, the timing of the blood draw during the hospitalization was not a pre-specified inclusion criteria.

## Exclusion criteria

Studies were excluded if they (i) only gave descriptive statistics of the biomarkers in one group of patients with COVID-19, (ii) were published only in abstract form, (iii) were non-English studies with no English translation, and/or (iv) were studies that reported measures of central tendency in groups other than those under consideration, i.e., died versus survived and critically ill versus not critically ill.

## Data extraction and assessment of study quality

For each study included, all data elements reported uniformly across most studies were extracted by two reviewers (AS and MM), cross-verified by a third (DD), and included in Table 1. The quality of each study and the risk of bias were evaluated using the Newcastle-Ottawa Quality Assessment Scale for nonrandomized studies.^17^ The following characteristics were assessed for sources of bias: (1) patient selection including definitions of exposure and representation of the larger population; (2) comparability of study groups and controlling for confounding factors by design or analysis; and (3) assessment and documentation of outcome including duration and loss of follow-up. Studies were graded as ‘poor’ if they met four or fewer of the nine criteria, ‘fair’ if they met five to six criteria, and ‘good’ if they met more than six criteria. For uniformity, all studies, regardless of study design, underwent quality bias assessment using the retrospective cohort scoring tool.

### Statistical methods

For the analysis of measures of central tendency of cardiac biomarkers in COVID-19 patients who died or were critically ill, median biomarker values were extracted for patients who died or were critically ill and patients who survived and/or were not critically ill, and the weighted mean differences (WMD) and 95% confidence interval (CI) between these two groups were calculated for each study. For the analysis of the risk of mortality with COVID-19 infection based on cardiac injury, event rates or univariate and multivariate odds ratios (OR) for mortality were extracted. For studies reporting Hazard ratio (HR) only, HR was adopted as the best estimate of OR. OR were transformed logarithmically, then standard error was calculated from Log OR and the corresponding 95% confidence interval (CI). The inverse variance method was used to achieve a weighted estimate of the combined overall effect. For all the analyses, we assessed the results for heterogeneity in our analysis by examining the forest plots and then calculating a Q statistic, which we then compared with the I^2^ index. We considered the presence of significant heterogeneity at the 5% level of significance (for the Q test) and values of I^2^ exceeding 56% as an indicator of significant heterogeneity.^18, 19^ We adopted the random effect model to pool WMD or effect sizes. All analyses were performed using Comprehensive Meta-Analysis version 3 (Biostat Inc., Englewood, NJ, USA).

For Meta-regression, we adopted a weighted regression random effect model and carried out a multivariate regression of pre-determined factors using comprehensive meta-analysis version 3. These variables were selected based on traditional factors shown to affect cardiovascular health and on data availability for the majority of the studies included. A two-sided P-value <0.05 was regarded as significant for all analyses. Data were represented as forest plots, and potential publication bias was assessed with the Egger test and represented graphically with Begg funnel plots of the natural log of the OR versus its standard error.

## Results

Twelve studies with 2,197 patients were included in the study. Through the initial database search, 718 studies were investigated for compliance with the inclusion criteria, of which 37 studies were ultimately chosen for consideration. Twenty-five studies were excluded from the meta-analysis for the following reasons: 1) 4 studies were descriptive and only reported biomarkers in all patients and did not compare critical/died versus not critical/alive patients. These studies did not de ne cardiac injury and did not assess OR for mortality with cardiac injury; 2) 19 studies did not report the biomarkers being studied; and 3) 2 studies reported biomarkers based on increased or normal D-dimers. Eleven studies were retrospective, and nine were single center (Table 1). Of the studies that reported time to blood sample collection, the average time reported was at the time of admission to 48 hours after admission. Of the studies that reported time to death or discharge, most of the studies reported it as the time from symptom onset to death or discharge. The average time for symptom onset to discharge was 27 days, and from symptom onset to death was 19 days. Two studies did not report median/mean cardiac biomarkers by predetermined patient groups,^20, 21^ but reported an OR for mortality based on cardiac injury. Five studies reported biomarkers based on whether the patients survived or died, ^20, 22–25^ five studies reported them based on whether the patient was critically ill or not. ^11–14, 26^ Of the latter studies, 4 categorized patients as sick based on the need for ICU admission, and 1 study based it on evidence of ARDS.^21^ Baseline demographics and comorbidities of patients included in the studies are shown in Table 2.

## Troponin

**Figure 2:**
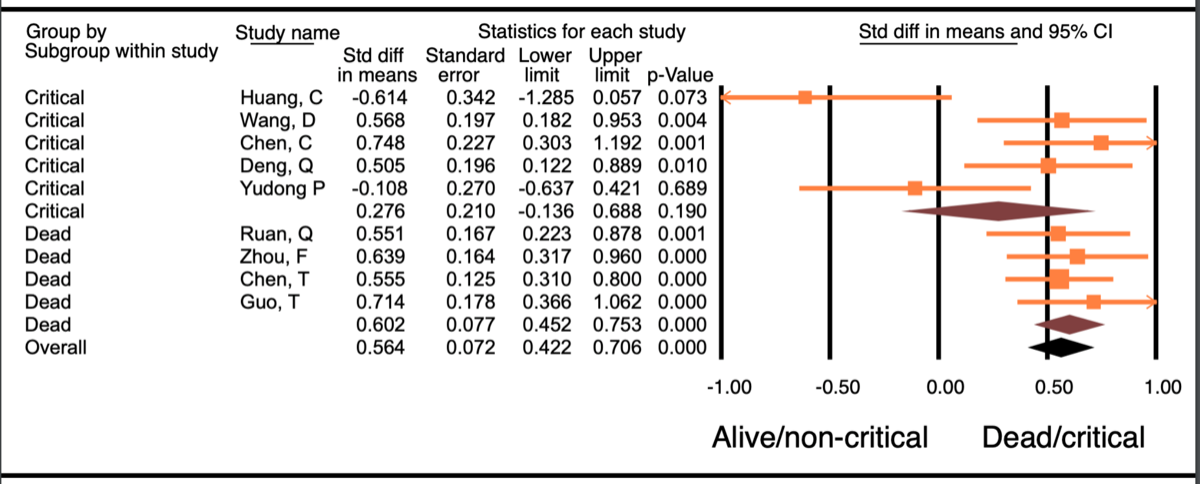
Forest plot of WMD in troponin between alive and/or not critically ill patients and dead and/or critically ill patients with COVID-19.

Nine studies with a total of 1,355 patients were included for this analysis. Five of the studies compared critically ill versus not critically ill patients, and the other 4 studies compared levels in dead versus alive patients. Results showed that patients who died or were critically ill had significantly higher troponin levels compared to patients who were alive or were not critically ill (WMD- 0.58, 95% CI 0.42–0.71, p<0.001). Sub-group analysis of the studies evaluating patients who died compared to patients who were alive showed that there was a significantly higher troponin level in those who died (WMD- 0.60, 95% CI 0.45–0.75, p<0.001). On the other hand, meta-analysis of studies comparing troponin levels in patients who were critically ill to those in patients who were not critically ill showed no difference in troponin levels (WMD- 0.28, 95% CI −0.14–0.69, p=0.19, Figure 2). The difference between these 2 groups of studies, i.e., studies that compared dead and alive and those that compared critically ill and not critically ill patients, was not significantly different (p-0.14).

The test for heterogeneity for the analysis of the 9 studies was an I^2^ of 58.5; therefore, a multivariate meta-regression analysis was performed with age, male gender, diabetes, hypertension, and cardiovascular disease. This analysis showed that hypertension (p=0.016, Supplemental Figure 2) and cardiovascular disease (p=0.001), (Supplemental Figure 3), contributed to the differences among studies in the reported troponin levels between the compared groups (R=1). Specifically, with increasing incidence of hypertension, there was a higher WMD in the troponin levels in patients who were dead or critically ill compared to levels in patients who were alive or not critically ill. The incidence of cardiovascular disease in the studies, on the other hand, was inversely related to the WMD of troponin between the groups.

### Risk of death based on biomarker proven cardiac injury

We included 4 studies that reported a hazard ratio for death or event rates based on cardiac injury. Of these studies, 1 study used CK-MB without providing a de nition.^26^ One study^16^ used troponin T and defined cardiac injury as troponin T levels above the 99^th^ percentile upper reference limit. Troponin I levels were used in two studies, and both studies defined cardiac injury as troponin I levels above the 99^th^ percentile upper reference limit. ^21, 24^

For the purpose of the meta-analysis, we extracted adjusted effect sizes, when available. In studies that did not report adjusted effect sizes, unadjusted OR was extracted. One study reported number of deaths and total sample size in each group, which was used in the analysis. Our analysis showed that cardiac injury was independently associated with significantly increased odds of mortality (OR 6.641, 95% CI 1.26–35.1, p=0.03).

## BNP

There were 5 studies with 835 patients that reported NT-proBNP. Three studies compared critically ill versus not critically ill patients, and the other 2 studies compared results from dead and alive patients. Analysis of these 5 studies did not show a significant difference in BNP levels in patients who died or were critically ill compared to levels in those who were alive or were not critically ill (WMD-0.35, 95% CI −0.15–0.86, p=0.17). Similarly, sub-group analysis showed that there was no significant difference in patients who died compared to patients who were alive (WMD-0.37, 95% CI −1.01–1.74, p=0.60) and patients who were critically ill and those who were not (WMD 0.35, 95% CI −0.20–0.89, p=0.21). (Supplemental Figure 1)

## Creatinine kinase (CK)

**Figure 3:**
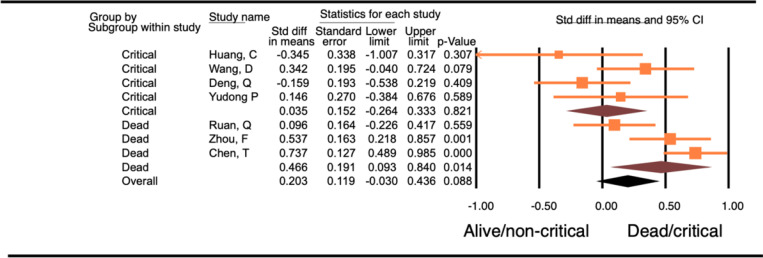
Forest plot of WMD in CK between alive and/or not critically ill patients and dead and/or critically ill patients with COVID-19.

Seven studies with a total of 1,018 patients were included in this analysis. Four of the studies compared critically ill versus not critically ill patients, and the other 3 studies compared CK levels in dead patients versus alive patients. There was no significant difference in the CK levels in patients who died or were critically ill and those in patients who were alive or were not critically ill (WMD 0.20, 95% CI −0.03–0.44, p=0.88). Sub-group analysis showed that there was a significantly higher CK level in patients who died (WMD-0.47, 95% CI 0.09–0.84, p=0.014) compared to patients who survived, whereas the patients who were critically ill did not have significantly higher CK levels compared to the patients who were not critically ill (WMD-0.04, 95% CI −0.26–0.33, p=0.82). (Figure 3) The difference between these 2 groups of studies (studies comparing dead versus alive and those that compared critically ill versus not critically ill patients) was not significantly different.

## D-dimer

Six studies with a total of 957 patients evaluated D-dimer levels in COVID-19 patients. Four studies compared critically ill versus not critically ill patients, and the other two compared levels from dead patients versus alive patients. Analysis of these six studies showed that patients who died or were critically ill had significantly hiFgher D-dimer levels compared to patients who were alive or were not critically ill (WMD OR 0.54, 95% CI 0.4–0.68, p<0.001). Sub-group analysis of studies evaluating patients who died compared to patients who were alive showed that there was a significantly higher D-dimer level in those who died (WMD-0.51, 95% CI 0.30–0.71, p<0.001). Similarly, critically ill patients had a significantly higher D-dimer level compared to the patients who were not critically ill (WMD-0.57, 95% CI 0.38–0.76, p<0.001). (Supplemental Figure 4)

## LDH

Eight studies with a total of 1,219 patients evaluated LDH levels in COVID-19 patients. Five of the studies compared critically ill versus not critically ill patients, and the other three compared levels from dead patients versus alive patients. Analysis of these eight studies showed that patients who died or were critically ill had significantly higher LDH levels compared to patients who were alive or were not critically ill (WMD-0.55, 95% CI 0.38–0.73, p<0.001). Sub-group analysis showed that there was a trend towards a higher LDH level in those who died (WMD-0.98, 95% CI −0.08–2.04, p=0.07) compared to those who were alive, and that critically ill patients had significantly higher LDH levels compared to patients who were not critically ill (WMD-0.54, 95% CI 0.36–0.72, p<0.001). The difference between these two groups of studies, dead versus alive and studies that compared critically ill versus not critically ill patients, was not significantly different. (Supplemental Figure 5)

## Discussion

To our knowledge, this is the first comprehensive meta-analysis focusing on the prognostic value of cardiac biomarkers in patients affected by the SARS-CoV-2 disease. Our study demonstrates that there exists a statistically significant association between disease severity and cardiac injury. Our analysis showed that troponin levels were higher among patients who died or were critically ill compared to those in patients who survived or were not critically ill. When analyzed by sub-groups, we found that troponin and CK were significantly elevated in patients with a fatal outcome compared to those who did not die, but was not significantly different in patients who were critically ill and those who were not. Interestingly, our analysis found that BNP levels were not significantly different in critically ill patients and/or patients who died, compared to not critically ill patients and/or patients who survived.

While pre-existing cardiac co-morbidities increase the risk of Acute COVID-19 Cardiovascular Syndrome (ACoVCS),^27,28^ recent evidence shows that SARS-CoV-2 can cause acute myocarditis-like illness in an otherwise healthy patient.^29^ The mechanism of cardiac injury caused by SARS-CoV-2 is not yet understood, but possible mechanisms include cytokine storm as the result of dysfunctional and uncontrolled immunological response, evidenced by increased levels of d-dimer, IL-6, CRP and LDH,^14, 30^ and primary cardiac injury caused by SARS-CoV-2. The first case of acute cardiac injury directly linked to myocardial localization of SARS-CoV-2, demonstrated by endomyocardial biopsy, was reported by Tavazzi et al.^31^ The viral envelope of SARS-CoV-2 is studded with glycoproteins called Spike proteins composed of two subunits. Subunit S1 has affinity for ACE2 receptors on the cell surface and Subunit S2 fuses with the cell membrane; acting together, these two proteins aid in the endocytosis of the viral particle.^32, 33^ Cardiac tissue has a significantly higher expression of ACE2 receptors and is therefore prone to direct cardiac injury in patients with COVID-19.^34, 35^

A recent study done by Liu et al. showed that increased levels of Angiotensin-2 (AT-2) in the plasma of patients infected with COVID-19 showed a linear association with the severity of the disease.^36^ SARS-CoV-2 reduces the expression of ACE2 due to internalization as shown in a study by Oudit et al., which demonstrated a marked decrease in ACE2 expression in the myocardium of mice infected with SARS-CoV-2. ^37^ ACE2 is responsible for the degradation of AT-2 into Ang 1–7, which is cardioprotective and has anti-inflammatory and anti-fibrotic properties.^38^ The loss of ACE2 shifts the balance towards an accumulation of AT-2, which has inflammatory and pro-thrombotic properties. AT-2 promotes vasoconstriction and increases the levels of Plasminogen Activator Inhibitor-1, which plays an active role in thrombogenesis and myocardial infarction.^38^ This shift in RAAS axis towards AT-2 could possibly explain high D-dimer levels, a biomarker that showed clearly increased levels in both critically ill patients and patients who died compared to levels in not critically ill and alive patients in our meta-analysis.

**Figure 4:**
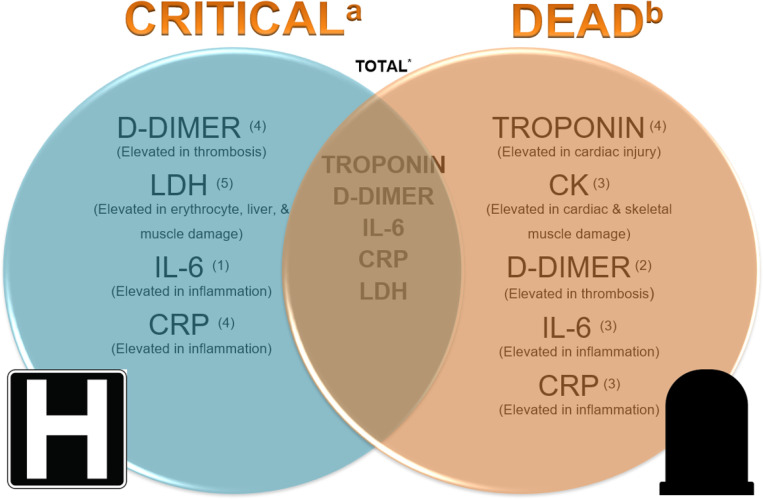
Biomarkers in COVID-19 patients. a Significant difference seen in biomarkers in COVID-19 patients who were critically ill b Significant difference seen in biomarkers in COVID-19 patients who died * Significant difference seen in biomarkers in COVID-19 patients who died and/or were critically ill Superscripts: total number of articles in each group Biomarkers studied: Troponin, BNP, CK, CRP, LDH, D-dimer, IL-6. BNP is not included in the diagram as it did not show a significant difference in the critically ill, the dead, nor the combined total of COVID-19 patients

Surprisingly, among the cardiac biomarkers (troponin, CK, and BNP) analyzed in this meta-analysis, while none of them had levels significantly higher in critically ill patients compared to levels in patients who were not critically ill, the levels of CK and troponin were significantly higher in patients who died compared to the levels in those who survived. Surprisingly, based on our meta-analysis, BNP levels did not differ in the critically ill or in those who were not critically ill, or in the patients who died and those who lived. In addition to ventricular stretch, BNP is a known marker for many other conditions, including lung disease, pulmonary hypertension, kidney dysfunction, and excessive cortisol levels. COVID-19 infection causes an assortment of these conditions in the different groups of patients, effectively neutralizing the differences between the groups. Troponin was the only cardiac biomarker that was higher in the combined analysis of critically ill and/or dead COVID-19 patients compared to not critically ill and/or alive patients. These results suggest that while there could be minimal changes in troponin in critically ill patients due possibly to non-cardiac reasons, significant elevation in COVID-19 patients may be an indication of direct myocardial injury, which in turn increases the risk of death, thereby making it a crucial tool in predicting death. Unfortunately, not many studies reported the MB isofraction of CK. In addition, the inflammatory biomarkers (IL-6 and CRP; see Supplementary Figures 6 and 7) and D-dimer levels from the same studies were significantly increased in the critically ill patients and the patients who died compared to levels in the control population, suggesting that it might be difficult to use these biomarkers to differentiate patients who are just critically ill from those at risk of dying. However, the difference in IL-6 (p-0.04) and CRP (p-0.04) levels between patients who died compared to levels in patients who survived was significantly higher than in patients who were critically ill and those who were not. Finally, LDH was significantly higher in critically ill patients compared to those who were not, but not in patients who died compared to those who survived. (See Figure 4)

## Limitations

Our study has several limitations. First, the critical and not critical groups included in our analysis are heterogenous, i.e, we defined critical as patients who required ICU admission as well as those who developed severe lung injury and ARDS. Similarly, the criteria for ICU admissions may differ from one hospital to another. Second, most studies were conducted in and published from China and therefore the generalizability of this information to other parts of the world and ethnicities is limited. The absence of details regarding the timing of measurement of biomarkers during the hospital stay limits the use of the results as an aid to the ‘predictability’ of death or critical illness. Last, the studies included in the meta-analysis evaluating the risk of mortality with cardiac injury varied in their definitions of cardiac injury.

## Supplementary Material

Supplement

Supplement

## Abbreviations

COVID–19: coronavirus 2019
WMD: weighted mean differences
CI: 95% confidence interval
OR: odds ratio
BNP: brain natriuretic peptide
CK: creatine kinase
LDH: lactate dehydrogenase
CRP: C-reactive protein
IL–6: interleukin 6
SARS-CoV–2: Severe Acute Respiratory Syndrome Coronavirus–2
