# Supplementary material for: Prognostic value of Cardiac Biomarkers in COVID-19 Infection: A Meta-analysis
Source: Res Sq. 2020 Jun 13:rs.3.rs-34729. Preprint. [Version 1] doi: 10.21203/rs.3.rs-34729/v1 (PMC7336705; doi:10.21203/rs.3.rs-34729/v1)
Supplement: Supplement [file COVIDmanuscriptSupplementalFigureLegends.pdf]

Prognostic value of Cardiac Biomarkers in COVID-19 Infection: A Meta-analysis.

Aakash Sheth*, MD, Malak Modi*, MD, Desiree' Dawson*, MD, Paari Dominic, MD

*Equal contributors

Supplemental Figure Legends:

e-Figure 1: Forest plot of WMD in BNP between alive and/or not critically ill patients and dead and/or critically ill patients with COVID-19.
e-Figure 2: Relationship between HTN and WMD in troponin levels in individual studies in a multivariate meta-regression model.
e-Figure 3: Relationship between CVD and WMD in troponin levels in individual studies in a multivariate meta-regression model.
e-Figure 4: Forest plot of WMD in D-dimer between alive and/or not critically ill patients and dead and/or critically ill patients with COVID-19.
e-Figure 5: Forest plot of WMD in LDH between alive and/or not critically ill patients and dead and/or critically ill patients with COVID-19.
e-Figure 6: Forest plot of WMD in IL-6 between alive and/or not critically ill patients and dead and/or critically ill patients with COVID-19.
e-Figure 7: Forest plot of WMD in CRP between alive and/or not critically ill patients and dead and/or critically ill patients with COVID-19.
e-Figure 8: Funnel plot for publication bias for studies reporting Troponin.
e-Figure 9: Funnel plot for publication bias for studies reporting CK.
e-Figure 10: Funnel plot for publication bias for studies reporting BNP.
e-Figure 11: Funnel plot for publication bias for studies reporting D-dimer.
e-Figure 12: Funnel plot for publication bias for studies reporting LDH.
e-Figure 13: Funnel plot for publication bias for studies reporting IL-6.
e-Figure 14: Funnel plot for publication bias for studies reporting CRP.

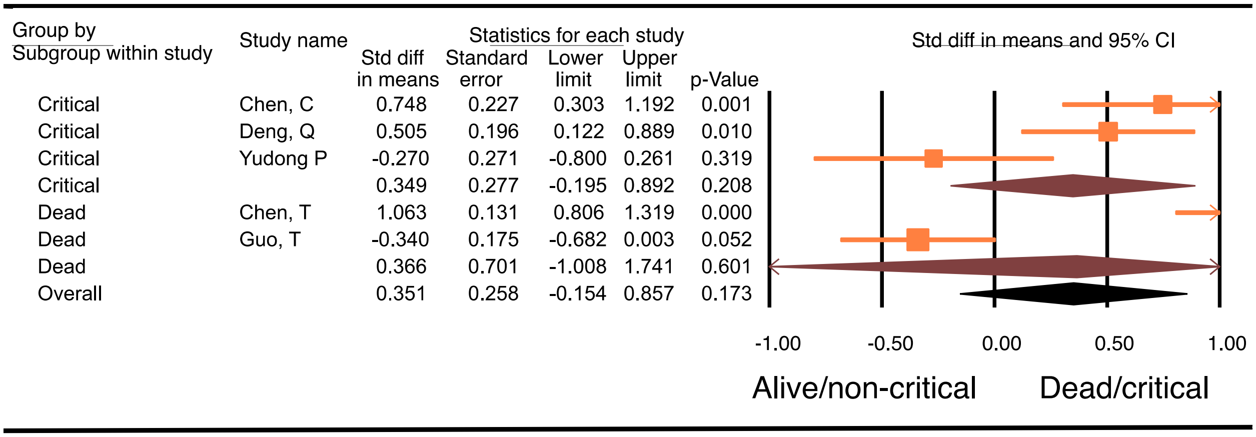

Supplemental Figure 1. Forest plot of WMD in BNP between alive and/or not critically ill patients and dead and/or critically ill patients with COVID-19.

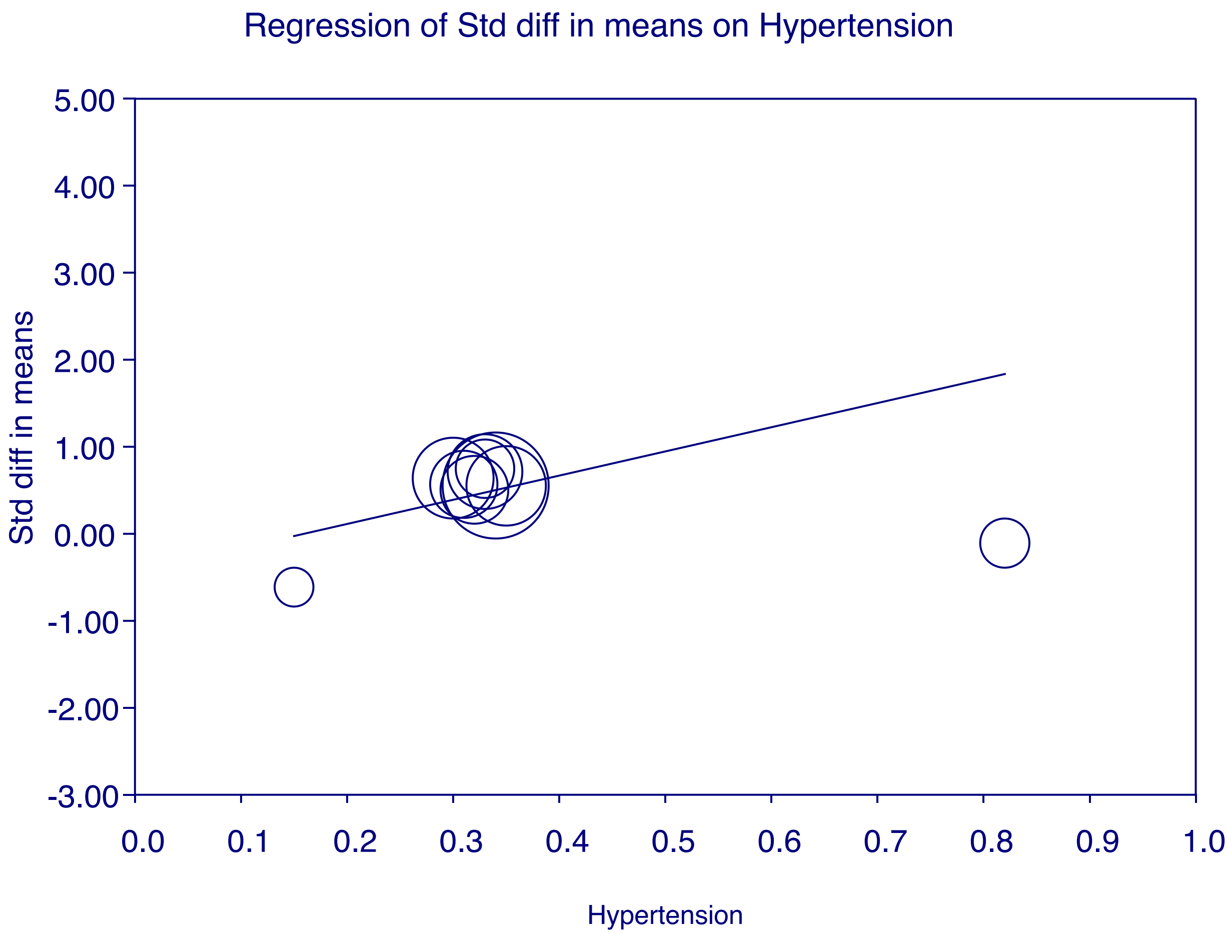

Supplemental Figure 2. Relationship between HTN and WMD in troponin levels in individual studies in a multivariate meta-regression model.

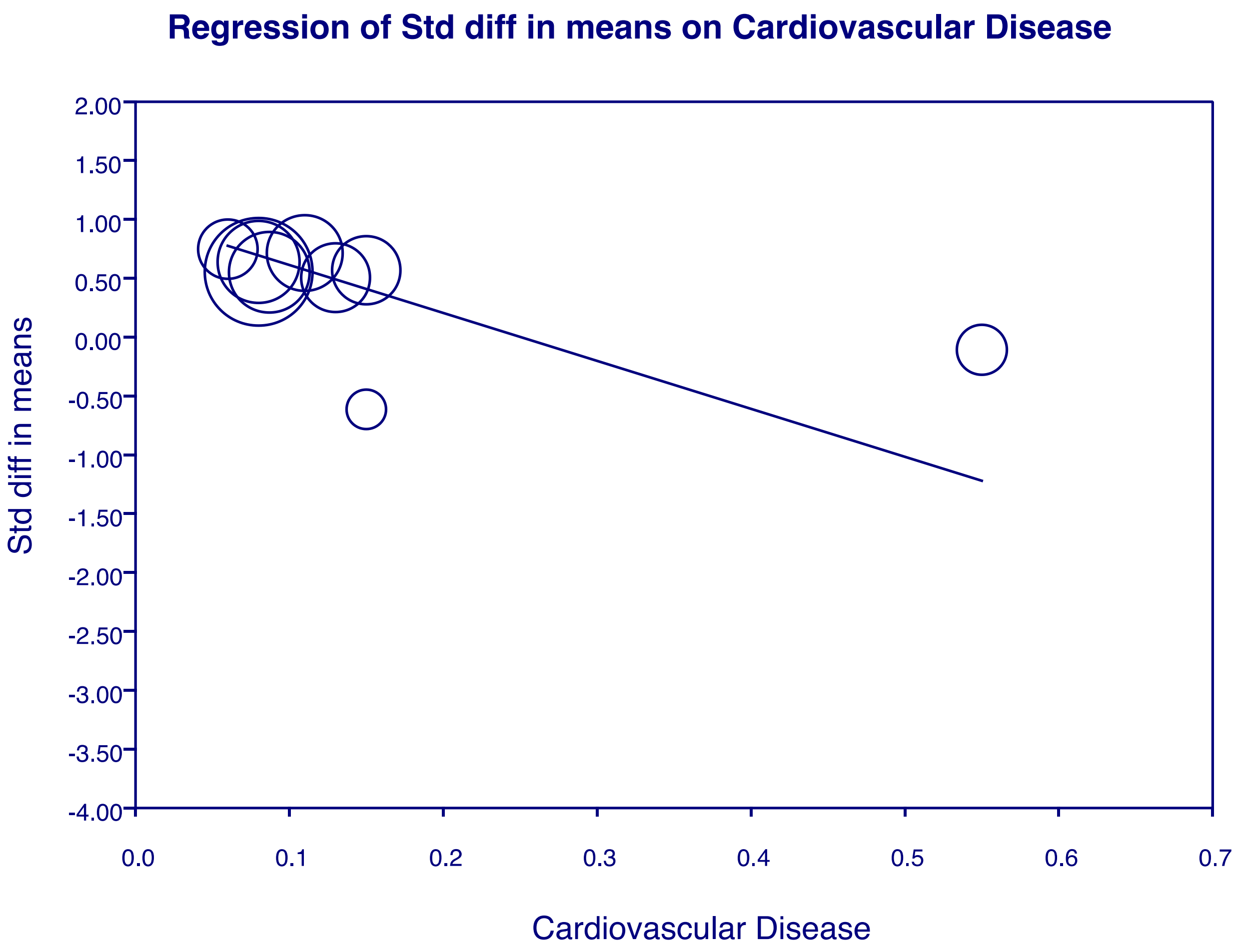

Supplemental Figure 3. Relationship between CVD and WMD in troponin levels in individual studies in a multivariate meta-regression model.

Group by Subgroup within study	Study name	Std diff in means	Standard error	Lower limit	Upper limit	p-Value
		Statistics for each study				
Critical	Huang, C	1.021	0.354	0.327	1.715	0.004
Critical	Wu, C	0.478	0.145	0.194	0.762	0.001
Critical	Wang, D	0.652	0.198	0.264	1.040	0.001
Critical	Deng, Q	0.505	0.196	0.122	0.889	0.010
Critical		0.566	0.097	0.377	0.756	0.000
Dead	Zhou, F	0.639	0.164	0.317	0.960	0.000
Dead	Chen, T	0.426	0.124	0.183	0.670	0.001
Dead		0.506	0.103	0.304	0.707	0.000
Overall		0.538	0.070	0.400	0.676	0.000

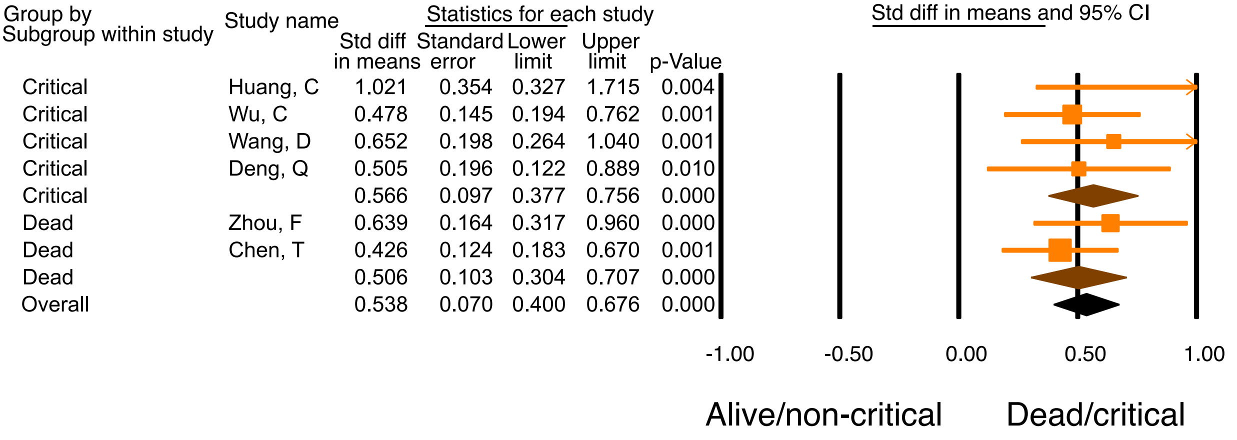

Supplemental Figure 4. Forest plot of WMD in D-dimer between alive and/or not critically ill patients and dead and/or critically ill patients with COVID-19.

Group by Subgroup within study	Study name	Std diff in means	Standard error	Lower limit	Upper limit	p-Value
Critical	Huang, C	1.015	0.354	0.321	1.708	0.004
Critical	Wu, C	0.478	0.145	0.194	0.762	0.001
Critical	Wang, D	0.652	0.198	0.264	1.040	0.001
Critical	Deng, Q	0.505	0.196	0.122	0.889	0.010
Critical	Yudong P	0.333	0.271	-0.198	0.864	0.219
Critical		0.540	0.091	0.361	0.718	0.000
Dead	Ruan, Q	0.289	0.165	-0.034	0.612	0.079
Dead	Zhou, F	0.639	0.164	0.317	0.960	0.000
Dead	Chen, T	2.013	0.150	1.719	2.307	0.000
Dead		0.982	0.540	-0.077	2.041	0.069
Overall		0.552	0.090	0.376	0.728	0.000

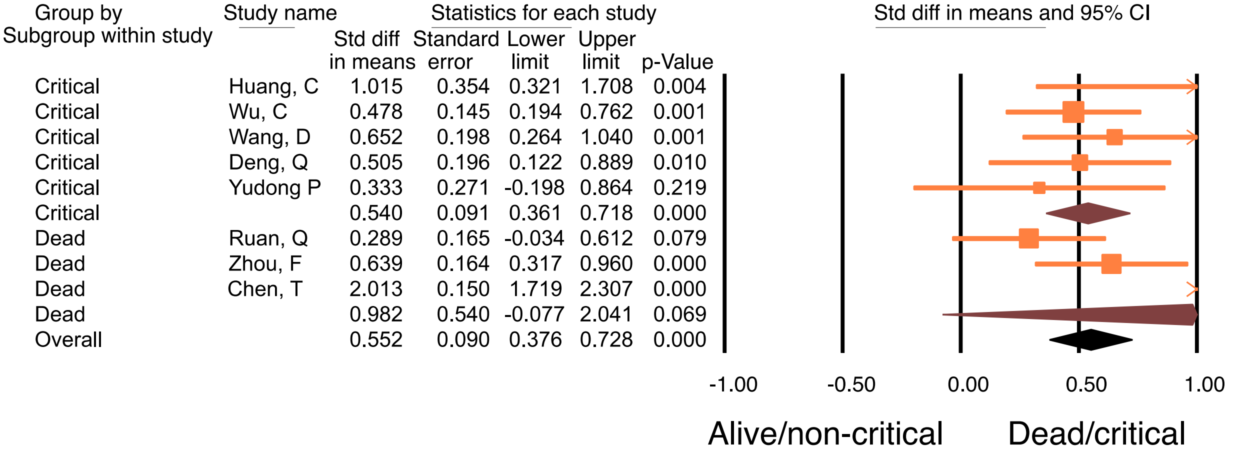

Supplemental Figure 5. Forest plot of WMD in LDH between alive and/or not critically ill patients and dead and/or critically ill patients with COVID-19.

Group by Subgroup within study	Study name	Std diff in means	Standard error	Lower limit	Upper limit	p-Value
Critical	Wu, C	0.313	0.144	0.031	0.595	0.030
Critical		0.313	0.144	0.031	0.595	0.030
Dead	Ruan, Q	0.551	0.167	0.223	0.878	0.001
Dead	Zhou, F	0.639	0.164	0.317	0.960	0.000
Dead	Chen, T	1.086	0.131	0.829	1.343	0.000
Dead		0.771	0.176	0.425	1.117	0.000
Overall		0.496	0.111	0.277	0.714	0.000

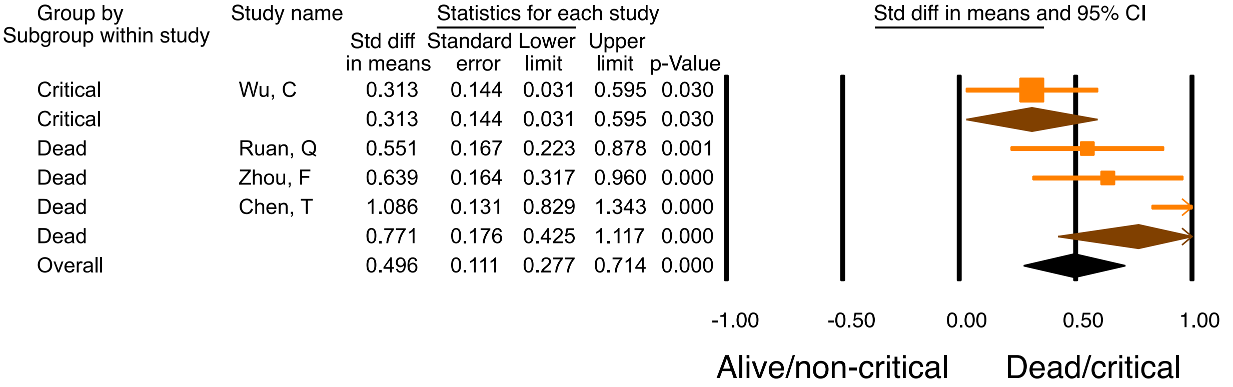

Supplemental Figure 6. Forest plot of WMD in IL-6 between alive and/or not critically ill patients and dead and/or critically ill patients with COVID-19.

Group by Subgroup within study	Study name	Std diff in means	Standard error	Lower limit	Upper limit	p-Value
Critical	Wu, C	0.478	0.145	0.194	0.762	0.001
Critical	Chen, C	0.748	0.227	0.303	1.192	0.001
Critical	Deng, Q	0.505	0.196	0.122	0.889	0.010
Critical	Yudong P	0.913	0.277	0.370	1.456	0.001
Critical		0.587	0.097	0.397	0.778	0.000
Dead	Ruan, Q	0.551	0.167	0.223	0.878	0.001
Dead	Deng, Y	1.502	0.151	1.206	1.798	0.000
Dead	Chen, T	1.600	0.140	1.324	1.875	0.000
Dead		1.222	0.320	0.595	1.849	0.000
Overall		0.641	0.093	0.459	0.823	0.000

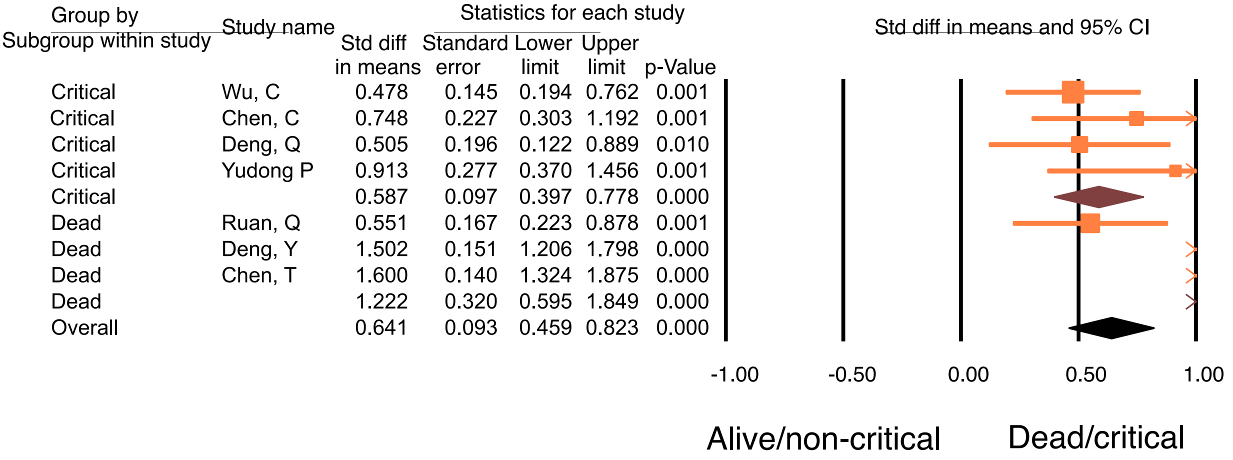

Supplemental Figure 7. Forest plot of WMD in CRP between alive and/or not critically ill patients and dead and/or critically ill patients with COVID-19.

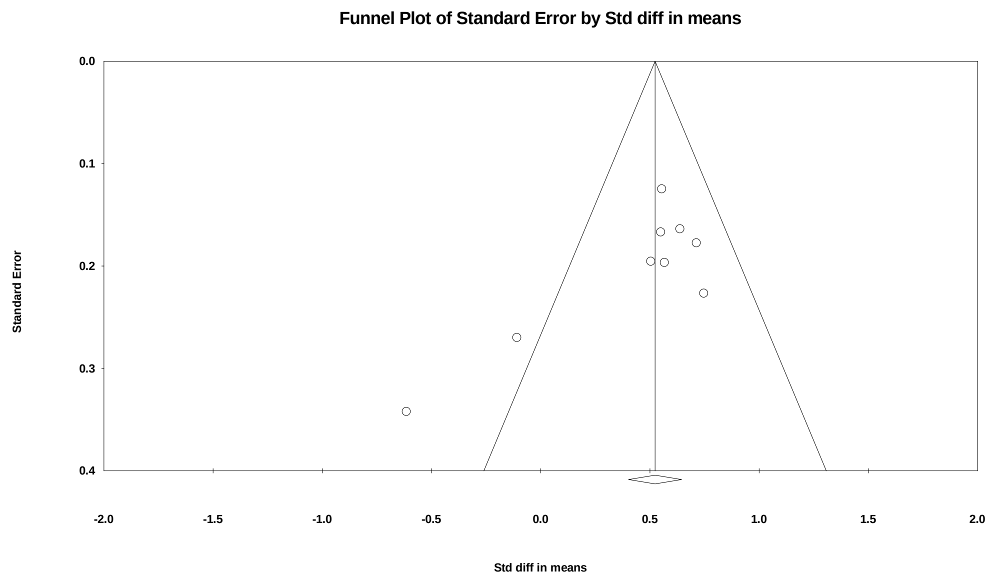

Supplemental Figure 8: Funnel plot for publication bias for studies reporting Troponin

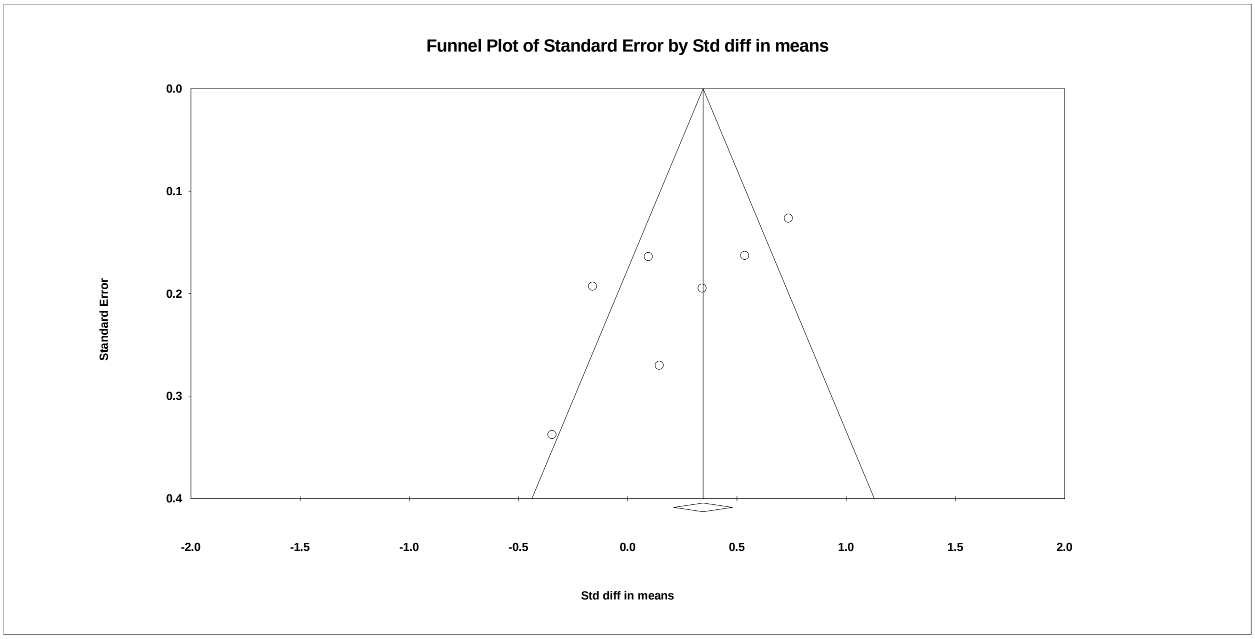

Supplemental Figure 9: Funnel plot for publication bias for studies reporting CK

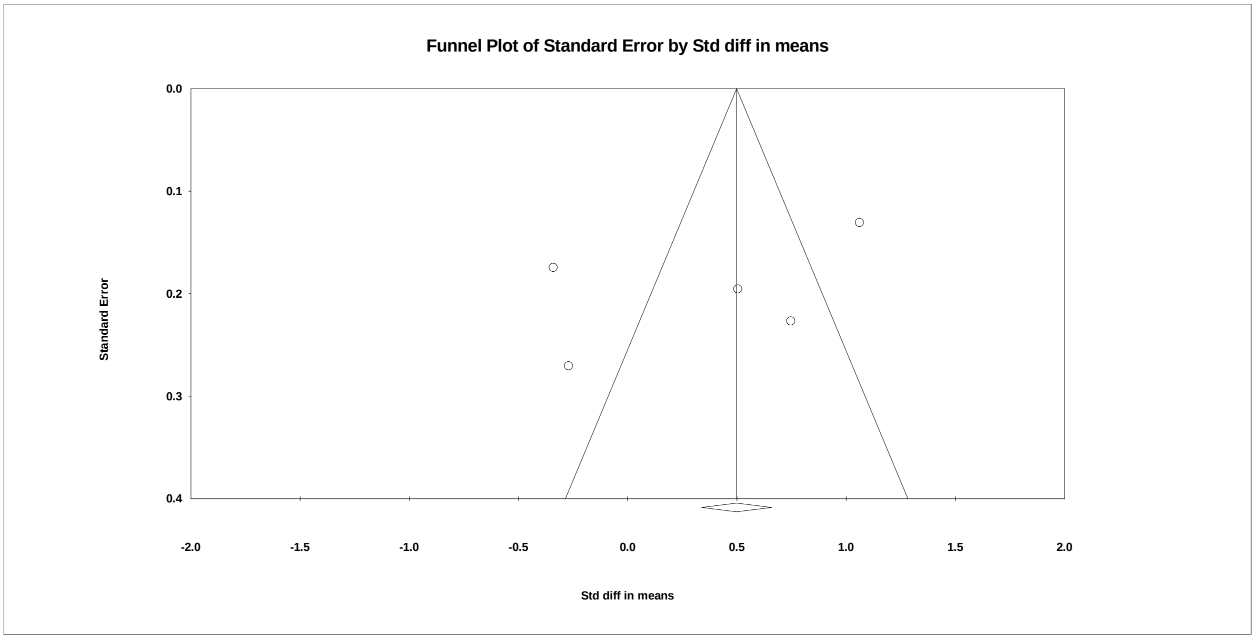

Supplemental Figure 10: Funnel plot for publication bias for studies reporting BNP

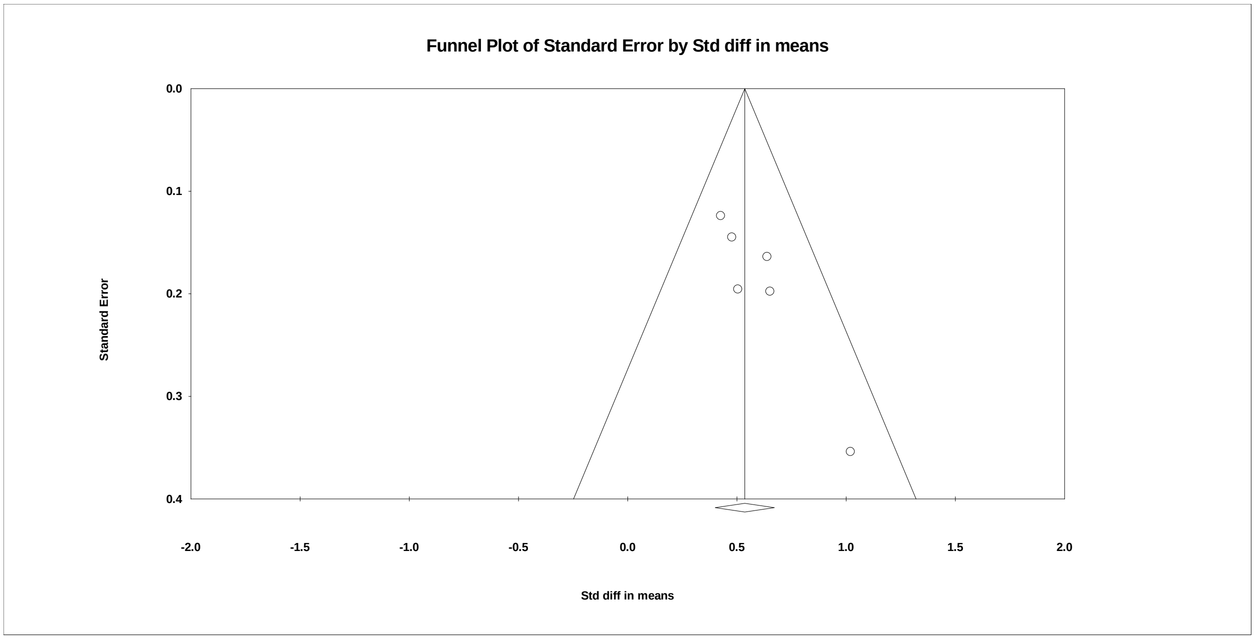

Supplemental Figure 11: Funnel plot for publication bias for studies reporting D-dimer

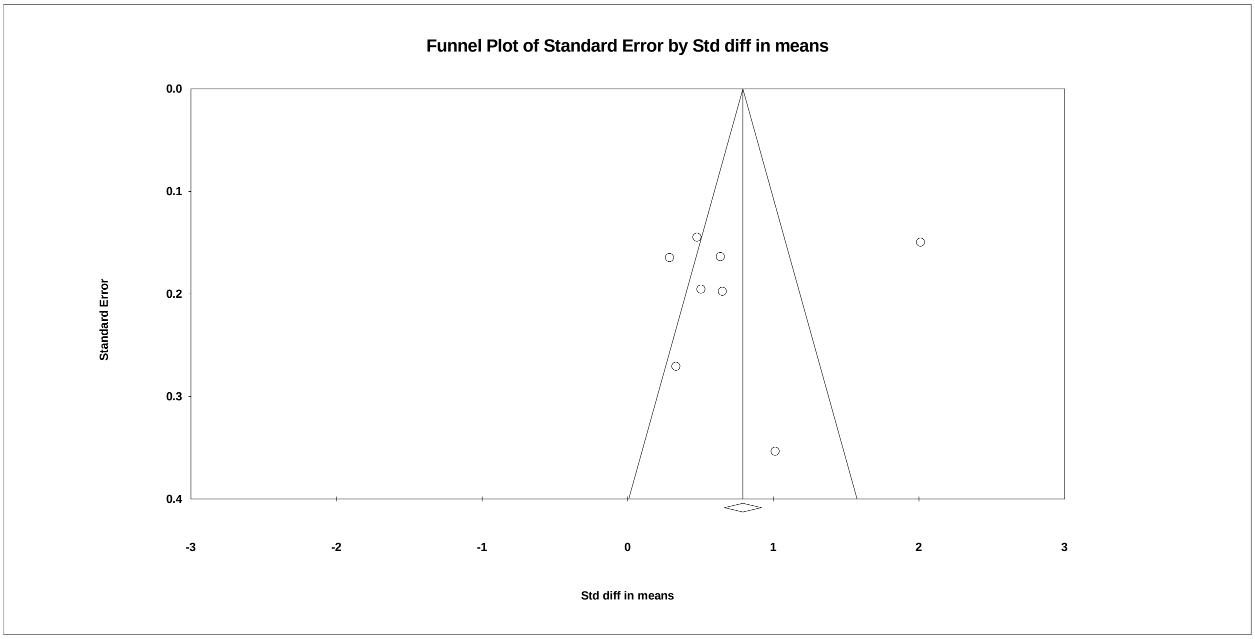

Supplemental Figure 12: Funnel plot for publication bias for studies reporting LDH

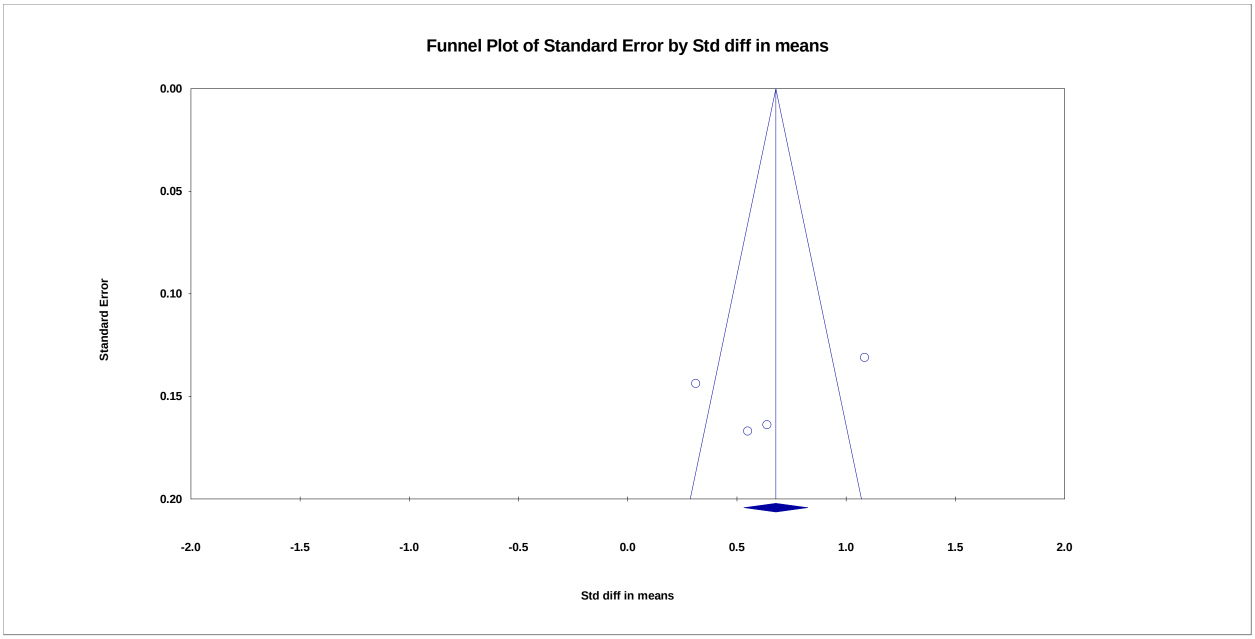

Supplemental Figure 13: Funnel plot for publication bias for studies reporting IL-6

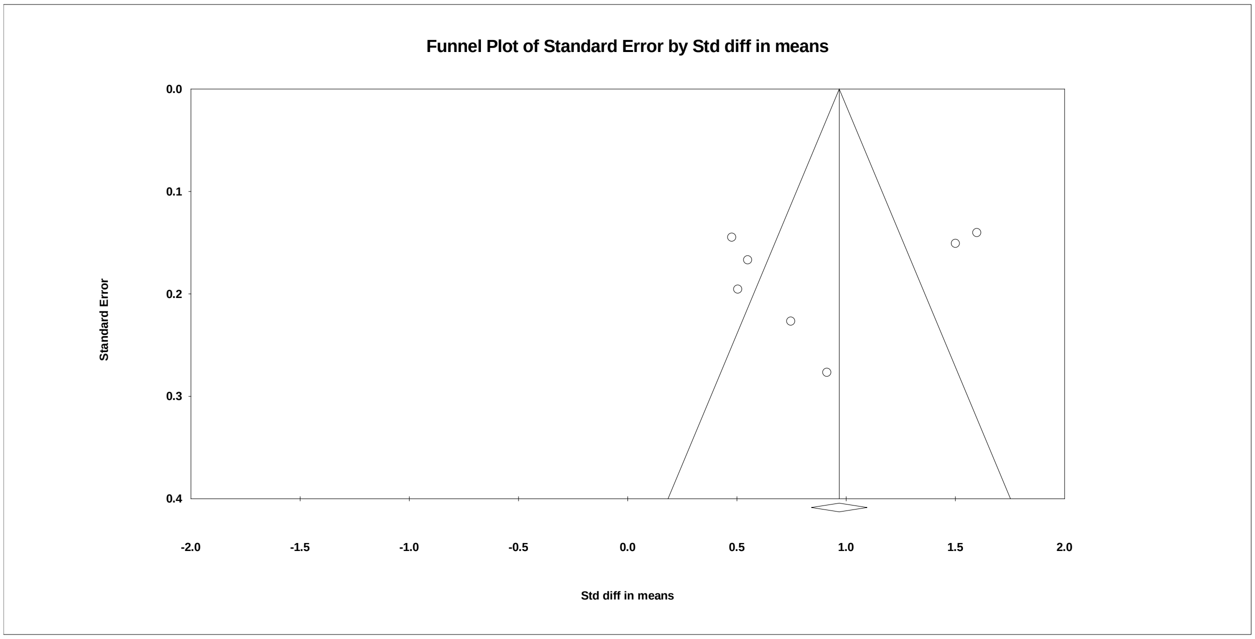

Supplemental Figure 14: Funnel plot for publication bias for studies reporting CRP
