# Supplementary material for: Prognostic value of Cardiac Biomarkers in COVID-19 Infection: A Meta-analysis
Source: Res Sq. 2020 Jun 13:rs.3.rs-34729. Preprint. [Version 1] doi: 10.21203/rs.3.rs-34729/v1 (PMC7336705; doi:10.21203/rs.3.rs-34729/v1)
Supplement: Supplement [file Tables.pdf]

Study	Region	Total # Patients	Study design	Time from symptom onset to Death (days)	Time from Admission to Death (days)	Time from Symptom onset to discharge (days)	Time from Admission to Discharge (days)	Troponin	CK	D-Dimer	LDH	NT-pro BNP	Quality Assessment Scale*
**Wang D et al., 2020** [11]	Wuhan, China	138	Single Center Retrospective Case series	--	--	--	--	☑	☑	☑	☑	☐	GOOD
**Chen C et al., 2020** [12]	Wuhan, China	150	Single Center Retrospective Cross sectional	--	--	--	--	☑	☐	☐	☐	☑	FAIR
**Deng Q et al., 2020** [13]	Wuhan, China	112	Single Center Retrospective Cohort	23 (15-39)		32 (IQR: 22-38)		☑	☑	☑	☑	☑	GOOD
**Huang C et al., 2020** [14]	Wuhan, China	41	Single Center Prospective Cohort	--	--	--	--	☑	☑	☑	☑	☐	GOOD
**Youdong P et al., 2020** [15]	Wuhan, China	112	Single Center Retropsective Cohort	--	--	--	--	☑	☑	☐	☑	☑	POOR
**Guo T et al., 2020** [20]	Wuhan, China	187	Single Center Retrospective Case series		With elevated TnT: 23.2 (8-41)			☑	☐	☐	☐	☑	GOOD
**Shi S et al., 2020** [21]	Wuhan, China	416	Single Center Retrospective Cohort		With cardiac injury: 6.3 (1-16) Without cardiac injury: 7.8 (1-23)			☑	☐	☐	☐	☑	GOOD
**Ruan Q et al., 2020** [22]	Wuhan, China	150	Multicenter Retrospective Case control	18.4 (5-48)				☑	☑	☐	☑	☐	FAIR
**Deng Y et al., 2020** [23]	Wuhan, China	225	Multicenter Retrospective Cohort	--	--	--	--	☐	☐	☐	☐	☐	FAIR
**Zhou F et al., 2020** [24]	Wuhan, China	191	Multicenter Retrospective Cohort	18.5 (15-22)		22 (18-25)		☑	☑	☑	☑	☐	GOOD
**Chen T et al., 2020** [25]	Wuhan, China	274	Single Center Retrospective Case series	16 (12-20)	5(3-9.3)	26 (21.8-29)	16 (14-19)	☑	☑	☑	☑	☑	GOOD
**Wu C et al., 2020** [26]	Wuhan, China	201	Single Center Retrospective Cohort	--	--	--	--	☐	☐	☑	☑	☐	GOOD

*Newcastle-Ottawa Quality Assessment Scale. Poor <4, Fair 5-6, Good >7

Table 1. Clinical characteristics and laboratory markers of patients affected by the Coronavirus Disease 2019 (COVID-19), in the included studies

AUTHOR	AGE (median)	MALE GENDER, n (%)	HYPERTENSION, n (%)	DIABETES, n (%)	CARDIOVASCULAR DISEASE, n (%)	COPD, n (%)
Ruan Q et al.,2020 [22]	57.7	102/150 (68)	53/150 (35)	26/150 (17)	14/150 (9)	3/150 (2)
Deng Y et al.,2020 [23]	54.1	124/225 (55)	59/225 (26)	27/225 (12)	18/225 (8)	25/225 (11)
Huang C et al.,2020 [14]	49	30/41 (73)	6/41 (15)	8/41 (20)	6/41 (15)	1/41 (2)
Wu C et al.,2020 [26]	52.4	128/201 (64)	38/201 (19)	22/201 (11)	8/201 (4)	NR
Wang D et al.,2020 [11]	56	75/138 (54)	43/138 (31)	14/138 (10)	21/138 (15)	4/138 (3)
Zhou F et al.,2020 [24]	56	119/191 (62)	57/191 (30)	36/191 (19)	15/191 (8)	6/191 (3)
Chen T et al.,2020 [25]	62	171/274 (62)	93/274 (34)	47/274 (17)	22/274 (8)	19/274 (7)
Chen C et al.,2020 [12]	58.9	84/150 (56)	50/150 (33)	20/150 (13)	9/150 (6)	NR
Deng Q et al.,2020 [13]	65	57/112 (51)	36/112 (32)	19/112 (17)	15/112 (13)	4/112 (4)
Guo T et al.,2020 [20]	58.5	91/187 (49)	62/187 (33)	28/187 (15)	21/187 (11)	4/187 (2)
Youdong P et al.,2020 [15]	62	53/112 (47)	92/112 (82)	24/112 (21)	62/112 (55)	NR
Shi S et al.,2020 [21]	64	205/416 (49)	129/416 (31)	58/416 (14)	46/416 (11)	12/416 (3)

Table 2: Baseline Demographics of patients with COVID-19.
NR- not reported
